# Clinico-pathological features of oropharyngeal squamous cell carcinomas in Malaysia with reference to HPV infection

**DOI:** 10.1186/s13027-018-0193-6

**Published:** 2018-06-11

**Authors:** Lee Fah Yap, Sook Ling Lai, Anthony Rhodes, Hans Prakash Sathasivam, Maizaton Atmadini Abdullah, Kin-Choo Pua, Pathmanathan Rajadurai, Phaik-Leng Cheah, Selvam Thavaraj, Max Robinson, Ian C. Paterson

**Affiliations:** 1 Faculty of Dentistry, University of Malaya, Kuala Lumpur, Malaysia; 2 Oral Cancer Research and Coordinating Centre, Faculty of Dentistry, University of Malaya, Kuala Lumpur, Malaysia; 3 School of Medicine, Taylor’s University, Subang Jaya, Selangor Malaysia; 4 Centre for Oral Health Research, Newcastle University, Newcastle-upon-Tyne, UK; 5 Ministry of Health, Kuala Lumpur, Malaysia; 6 Faculty of Medicine and Health Sciences, University Putra Malaysia, Serdang, Malaysia; 7 Penang General Hospital, Penang, Malaysia; 8 Subang Jaya Medical Centre, Subang Jaya, Selangor Malaysia; 9 Faculty of Medicine, University of Malaya, Kuala Lumpur, Malaysia; 10 Head and Neck Pathology, Dental Institute, King’s College London, London, UK

**Keywords:** Oropharyngeal, Squamous cell carcinoma, p16, Human papillomavirus, Malaysia

## Abstract

**Background:**

The incidence of oropharyngeal squamous cell carcinoma (OPSCC) has been rising in Western countries and this has been attributed to human papillomavirus (HPV) infection. p16 expression is a marker for HPV infection and p16 positive OPSCC is now recognized as a separate disease entity. There are only limited data available regarding HPV-related OPSCC in Asian countries and no data from Malaysia.

**Methods:**

We identified 60 Malaysian patients with OPSCC over a 12-year period (2004–2015) from four different hospitals in two major cities, Kuala Lumpur and Penang. The detection of HPV was carried out using p16 immunohistochemistry and high risk HPV DNA in situ hybridisation.

**Results:**

Overall, 15 (25%) tumours were p16 positive by immunohistochemistry, 10 of which were also positive for high risk HPV DNA by in situ hybridisation. By comparison, a matched cohort of UK patients had a p16 positive rate of 49%. However, between 2009 and 2015, where cases were available from all four hospitals, 13 of 37 (35%) cases were p16 positive. In our Malaysian cohort, 53% of patients were of Chinese ethnicity and 80% of the p16 positive cases were found in these patients; no Indian patients had p16 positive disease, despite representing 35% of the total cohort.

**Conclusion:**

The proportion of OPSCCs associated with HPV in Malaysia appears to be lower than in European and American cohorts and could possibly be more prevalent amongst Malaysians of Chinese ethnicity. Further, our data suggests that the burden of HPV-related OPSCC could be increasing in Malaysia. Larger cross-sectional studies of Malaysian patients are required to determine the public health implications of these preliminary findings.

## Background

The profile of head and neck squamous cell carcinoma (SCC) has changed over the past few decades with increased rates of oropharyngeal SCC (OPSCC) having been documented in Europe and the USA [1]. Although risk factors such as tobacco and alcohol consumption are still important for the development of OPSCC, it has become apparent that oncogenic human papillomavirus (HPV) is also an important aetiological agent [2]. HPV is thought to account for the relatively recent increase in OPSCC, with data from Sweden and the USA indicating that 70–80% of OPSCC are HPV positive [3, 4]. Patients with HPV positive tumours are typically non-smokers and have a low consumption of alcohol. Sexual behaviour, such as early age of sexual debut and increasing numbers of sex partners appears to correlate with HPV-related OPSCC [5, 6]. Furthermore, HPV-related OPSCC patients have better survival rates than those with HPV negative tumours [6–12].

p16 immunohistochemistry is used clinically as a surrogate marker for oncogenic HPV infection in OPSCCs and in 2017 UICC and AJCC TNM8 classification assigned p16 positive OPSCC a separate staging system [13]. In developed countries, routine testing for p16 is now recommended for all patients with OPSCC as well as those with metastatic SCC of unknown primary in the head and neck region [14, 15].

At this point in time, there is a lack of accurate epidemiological and clinico-pathological data on the burden of HPV-related OPSCC in Malaysia, as reported by the HPV Information Centre [16]. Two previous studies on HPV-related HNSCCs in Malaysian patients were performed on mostly oral SCC specimens and as such are not representative of the burden of HPV-related OPSCC in Malaysia [17, 18]. Therefore, the aim of this study was to measure the proportion of Malaysian patients with p16 positive OPSCC and to examine the clinico-pathological features.

## Methods

### Patients and specimens

To ensure that the cohort was representative of the Malaysian population and to limit bias, cases were obtained from four different hospitals in two major cities. Cases were identified by searching pathology databases for SCCs coded as oropharynx, tonsil and soft palate. The patients were identified over a 12-year period (2004–2015). Formalin-fixed paraffin-embedded (FFPE) tissue blocks were obtained from the relevant pathology tissue archives and clinico-pathological information were obtained from clinical databases and review of medical records. All patient information was anonymised. This study had ethical approval from the relevant institutional medical research and ethics boards (Reference Numbers: NMRR-12-13,577; UMMC 20164–2341; SDMC 201211.3).

A matched UK cohort of patients was identified from an existing database at Newcastle-upon-Tyne Hospitals NHS Foundation Trust. Cases were matched by year of diagnosis, age at diagnosis (±10 years) and sex. Results for p16 immunohistochemistry staining were obtained from patient records. The study had favourable ethical opinion from the National Research Ethics Service Committee North East, Sunderland (REC reference: 11/NE/0118).

### HPV testing

#### p16 immunohistochemistry

p16 immunohistochemistry (IHC) was performed using a proprietary kit (CINtec Histology, Roche mtm laboratories AG, Germany) on a Ventana Benchmark Autostainer (Ventana Medical Systems Inc., USA). Normal tonsil was used as a negative control and OPSCC with high p16 expression was used as a positive control. p16 staining was assessed as positive when there was strong and diffuse nuclear and cytoplasmic staining present in greater than 70% of the malignant cells [19, 20].

#### High risk HPV DNA in situ hybridisation

HR- HPV DNA in-situ hybridisation (HR-HPV ISH) was carried out using proprietary reagents (Inform HPV III Family 16 Probe (B), Ventana Medical Systems Inc., USA) on a Benchmark Autostainer (Ventana Medical Systems Inc., USA). The Inform HPV III Family 16 Probe (B) detects high risk genotypes HPV-16, − 18, − 31, − 33, − 35, − 39, − 45, − 51, − 52, − 56, − 58 and − 66. Three control samples were used: FFPE CaSki cells (HPV-16 positive; 200–400 copies per cell), HeLa cells (HPV-18 positive; 10–50 copies per cell) and C-33A (HPV negative; Ventana Medical Systems Inc., USA). The HR- HPV ISH test was scored as positive if there was any blue reaction product that co-localised with the malignant cells [21].

### Statistical analysis

Statistical analysis was performed using SPSS for Windows (version 21.0; SPSS Inc., USA). p16 positive and negative cases and patient characteristics were compared using independent t tests and Pearson’s Chi Square test. Results were considered significant at the 5% level (*p* < 0.05).

## Results

### HPV status of OPSCCs

**Fig. 1:**
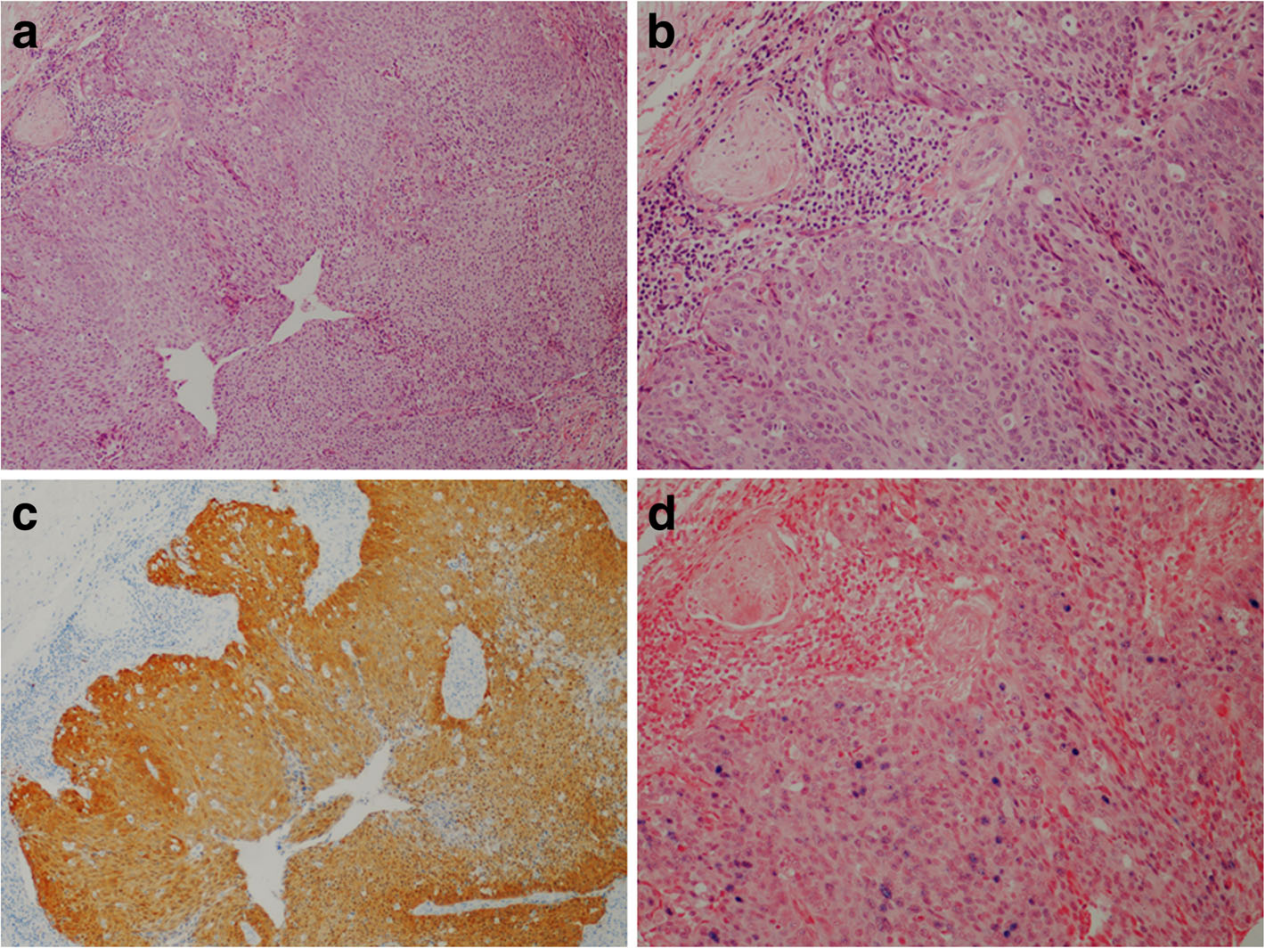
HPV-related oropharyngeal squamous cell carcinoma (**a** & **b**; H&E stain) showing high levels of p16 expression by immunohistochemistry (**c**) and evidence of high risk HPV DNA by in situ hybridisation (**d**)

We tested OPSCCs from 60 patients identified from four hospitals. 15 (25%) cases showed p16 expression, but only 10 of the p16 positive tumours (67%) showed evidence of high risk HPV DNA by in situ hybridisation (Table 1; Fig. 1). To make a more representative comparison, we identified samples collected from all four hospitals within the same period of time. A total of 37 samples were collected between 2009 and 2015 and 35% (13 of 37) of these cases were p16 positive.

**Table 1:** Demographics of Malaysian patients with p16 positive OPSCC

Case	Year of diagnosis	Age	Sex	Ethnicity	p16 IHC	HR-HPV ISH
1	2005	NK	NK	Malay	+	–
2	2006	88	F	Chinese	+	+
3	2009	41	F	Chinese	+	–
4	2010	64	F	Chinese	+	+
5	2012	56	M	Chinese	+	–
6	2012	56	M	Chinese	+	+
7	2012	67	F	Chinese	+	+
8	2013	NK	NK	Chinese	+	+
9	2013	74	M	Malay	+	+
10	2013	36	M	Malay	+	+
11	2013	53	M	Chinese	+	+
12	2013	70	M	Chinese	+	–
13	2014	72	M	Chinese	+	–
14	2014	72	F	Chinese	+	+
15	2015	54	M	Chinese	+	+

### Clinico-pathological profile of patients

Complete demographic data were available for 54 patients; the age and sex for six of the patients were not available. The clinico-pathological profiles of the patients are shown in Table 2. The mean age of patients was 65.44 years (± 12.16) at diagnosis and ranged from 36 to 93 years-old. There was no statistically significant difference in age between patients who had p16 negative and p16 positive OPSCC (*p* = 0.214). However, the two youngest (36 and 41 years of age) patients in the cohort had p16 positive disease. The overall male to female ratio was 2.4:1 and the ratio was similar in p16 negative cases (2.7:1), however, the ratio was lower in p16 positive cases (1.6:1). Most patients in the cohort were of Chinese ethnicity (53.3%) followed by Indians (35.0%). All the Indian patients had p16 negative disease, whilst 80% of the HPV positive cases were Chinese; this finding was statistically significant (*p* = 0.004). Overall, most of the OPSCC were classified as moderately differentiated SCC (40%) and this was similar for p16 negative cases (47%). By contrast, the majority of p16 positive cases (60%) were poorly differentiated SCC, which was statistically significant (*p* = 0.016).

**Table 2:** Clinico-pathological characteristics of Malaysian patients with OPSCC

	All patients (*n* = 60; 100%)	p16 negative (*n* = 45; 75%)	p16 positive (*n* = 15; 25%)	*p*-value
Age at diagnosis (years; *n* = 54)				
Mean (±SD)	65.44 (±12.16)	66.61 (±11.36)	61.77 (±14.28)	^a^0.214
Sex (*n* = 54)				
Male	38 (70.4%)	30 (73.2%)	8 (61.5%)	^b^0.493
Female	16 (29.6%)	11 (26.8%)	5 (38.5%)	
Ethnicity (*n =* 60)				
Malay	7 (11.7%)	4 (8.9%)	3 (20%)	^b^0.004
Chinese	32 (53.3%)	20 (44.4%)	12 (80%)	
Indian	21 (35.0%)	21 (46.7%)	0 (0%)	
Broder’s grade (*n* = 60)				
WD	16 (26.7%)	13 (28.9%)	3 (20.0%)	^b^0.016
MD	24 (40.0%)	21 (46.7%)	3 (20.0%)	
PD	17 (28.3%)	8 (17.8%)	9 (60.0%)	
Others	3 (5.0%)	3 (6.7%)	0 (0%)	

^a^Independent sample’s t-test

^b^Pearson’s Chi-Square test

*WD* Well differentiated, *MD* Moderately differentiated, *PD* Poorly differentiated

### Comparison with a matched UK cohort

A UK cohort of patients with OPSCC was used as a comparator. The Malaysian patients were matched with UK patients by year of diagnosis, age at diagnosis (±10 years) and sex. Fifty-one patients could be matched between the cohorts; six Malaysian patients had incomplete demographic data and three had data that could not be matched with a UK counterpart. The matched UK patients had a p16 positive rate of 49%, which was double that of the Malaysian patients (24%).

## Discussion

In previous years, SCCs of the oral cavity and oropharynx were often grouped together and thought of as being a single disease entity [22]. However, this has changed in recent years due to the recognition of HPV as a major aetiopathogenic agent in a subset of OPSCC. HPV positive OPSCC is now recognised as a clinico-pathologically unique form of HNSCC with distinct demographic, clinical and morphological features, as well as being associated with improved clinical outcomes [7, 23–26]. These findings have prompted the changes to oropharyngeal tumours in the 2017 edition of the WHO Classification of Head and Neck Tumours. The new edition has divided tumours of the oral cavity and oropharynx into different chapters and has also sub-classified OPSCC according to HPV status [27]. Furthermore, the UICC and AJCC have recently recommended new clinical and pathological staging systems for p16 positive OPSCC, which reflects the improved prognosis of the disease [13, 28].

The prevalence of p16 positive OPSCC in the Malaysian cohort was half that of a matched UK cohort (25% vs. 49%). The matched UK cohort was representative of a larger (*n* = 1529) multicentre prevalence study carried out in the UK demonstrating that OPSCC had a p16 positive rate of 54% [29]. Higher rates have also been reported elsewhere in Europe and America (35% vs 80%) [1]. A recent meta-analysis looking at the burden of HPV related head and neck cancers in the Asia Pacific region reported an overall prevalence of 40.53% for oropharyngeal cancers [30]. However, there were considerable differences in the rates between regions and countries; the region with the highest prevalence was Oceania (49.32%) and the country with the lowest prevalence was China (9.50%) [30]. Our findings taken for cases diagnosed between 2009 and 2015 (35%) are comparable to the reported rates in East and South Asian regions (25.8 and 38.7% respectively) and Singapore (42%) [31]. In the present study, 80% of the p16 positive cases were from patients of Chinese ethnicity, whilst all the Indian patients had p16 negative OPSCC. This finding could be particularly relevant because according to the 2010 Population and Housing Census of Malaysia, the Chinese account for only 24.6% of the total population [32]. Although this potentially alarming finding needs to be confirmed in a larger cohort, further research specifically into risk factors that predispose to HPV infection in the oropharynx are warranted in different populations.

Most epidemiological and clinical studies have indicated that patients with HPV positive OPSCC are relatively younger than patients with HPV negative disease [6, 30, 33–35], although our findings do concur with those studies with the mean age of HPV positive patients being slightly lower than the mean age of HPV negative patients, the finding was not statistically significant. According to the recent meta-analysis by Shaikh et al. [30], the prevalence of HPV associated head and neck cancer is higher amongst males, however the studies involved in the meta-analysis involved small study samples as well as unequal gender distributions [30]. The findings from our study are also similar with the findings of the meta-analysis with a slight male predilection.

The recent WHO 2017 edition has discouraged histologic grading of HPV positive OPSCC as there is insufficient evidence to correlate histopathological grading with clinical behaviour and outcomes [27]. All the cases in our study were diagnosed well before the release of the new WHO guideline and were based on previous guidelines that grouped oropharyngeal SCCs with oral cavity SCCs. The majority of HPV positive cases were graded as being poorly differentiated SCCs (70%). This “high grade” histopathologic category was probably based on the non-keratinized, immature and basaloid appearance of the tumour cells. However, such grading may not be accurate as HPV positive OPSCCs mostly arise from the epithelial lining of the tonsillar crypts and therefore retain the non-keratinizing and basaloid appearance of this epithelium.

The disparity between p16 positive rates (25%) and the detection of HR-HPV DNA (17%) is a consequence of the low sensitivity of HR-HPV DNA in situ hybridisation, which has been reported previously [36]. The relatively high rate of p16 positive, high risk HPV DNA ISH negative cases (5 of 15) is likely to reflect pre-analytical variables related to tissue fixation, processing and storage conditions. The HPV tests were conducted in an ISO15189:2012 accredited UK pathology laboratory, where such cases represent around 10% of OPSCCs tested [21]. The use of polymerase chain reaction and RNA-based in situ hybridisation has been shown to increase the detection rates and mitigate against false negative results [37].

## Conclusion

The results from this study suggest that the occurrence of HPV-related OPSCC in Malaysia may not be as high as those reported in developed nations such as the UK and the USA, but the proportion of OPSCCs that are HPV positive appears to be increasing, particularly in patients of Chinese ethnicity. Further studies will be required to determine how these observations might impact upon Malaysian communities and the national healthcare system in the future.

## Abbreviations

HPV: Human papillomavirus
ISH: In-situ hybridisation
OPSCC: Oropharyngeal squamous cell carcinoma
SCC: Squamous cell carcinoma
